# SARS-CoV-2 infection in pregnant women and incidence of thromboembolic disease: an analysis of the Covid-19-Related Obstetric and Neonatal Outcome Study (CRONOS) in Germany

**DOI:** 10.1007/s00404-025-08007-5

**Published:** 2025-03-25

**Authors:** Ulrich Pecks, Michael K. Bohlmann, Kristin Andresen, Johanna Büchel, Catharina Bartmann, Magdalena Sitter, Anastasia Tihon, Peter Kranke, Achim Wöckel, Regina Hollweck, Iris Dressler-Steinbach, Susanne Gruessner, Teresa M. Gruber, Teresa Eichinger, Jula Manz, Ina M. Ruehl, Angela Lihs, Anna-Lena Biermann, Lea M. Bauerfeind, Kathleen M. Oberste, Babett Ramsauer, Eveline Russe, Susanne Schrey-Petersen, Filiz Markfeld Erol, Cahit Birdir, Lisa Kaup, Gregor Seliger, Christine Morfeld, Martin A. Berghaeuser, Manuela F. Richter, Peter Jakubowski, Birgit Linnemann, Werner Rath

**Affiliations:** 1 Department of Obstetrics and Gynecology, Germany AND Institut of Midwifery, University Hospital of Wuerzburg, Würzburg, Germany; 2 Department of Obstetrics and Gynecology, St. Elisabethen-Krankenhaus Lörrach, Lörrach, Germany; 3 Department of Obstetrics and Gynecology, University Hospital of Schleswig-Holstein, Kiel, Germany; 4 Department of Obstetrics and Gynecology, University Hospital of Wuerzburg, Würzburg, Germany; 5 Department of Anesthesiology, University Hospital of Wuerzburg, Würzburg, Germany; 6 Novustat GmbH, Roosstr. 43, 8832 Wollerau, Switzerland; 7 Department of Obstetrics, Charité, Berlin, Germany; 8 Department of Gynecology and Obstetrics, Klinikum Wilhelmshaven, Wilhelmshaven, Germany; 9 Department of Gynecology, Obstetrics and Gynecological Endocrinology, Johannes Kepler University Linz, Linz, Austria; 10 Department of Obstetrics and Gynecology, Klinikum Darmstadt GmbH, Darmstadt, Germany; 11 Department of Obstetrics, Red Cross Hospital “Taxisstrasse”, Munich, Germany; 12 Department of Gynecology and Obstetrics, City Hospital Sindelfingen-Boblingen, Boblingen, Germany; 13 Department of Obstetrics and Gynecology, Hannover Medical School, Hannover, Germany; 14 Department of Obstetrics and Gynecology, LMU University Hospital, Munich, Germany; 15 Department of Obstetrics and Gynecology, University Hospital Muenster, Muenster, Germany; 16 Department of Obstetrics, Vivantes-Klinikum Neukölln, Berlin, Germany; 17 Department of Obstetrics and Gynecology, St. Elisabeth-Hospital Lörrach, Lörrach, Germany; 18 Department of Obstetrics, University of Leipzig, Leipzig, Germany; 19 Clinic for Gynaecology and Obstetrics, University Medical Center Freiburg, Freiburg, Germany; 20 Department of Obstetrics and Gynecology, University Clinic of Dresden, Dresden, Germany; 21 Department of Obstetrics and Gynecology, Dr Geisenhofer Clinic for Gynecology and Obstetrics, Munich, Germany; 22 Outpatient centre for women’s health, fertility and pregnancy, University Medicine Halle, Halle (Saale), Germany; 23 Perinatal Care Center, Diakovere Hannover, Hannover, Germany; 24 Department of Paediatrics, Florence-Nightingale Hospital, Duesseldorf, Germany; 25 Neonatology, AUF DER BULT-Children’s and Youth Hospital, Hannover, Germany; 26 Department of Obstetrics, University Hospital of Tuebingen, Tuebingen, Germany; 27 Center for Cardiology and Angiology, Johannes-Gutenberg-University, Mainz, Germany

**Keywords:** Stillbirth, Blood transfusion, Maternal death, Invasive ventilation, Heparin

## Abstract

**Purpose:**

The aim of the present study was to quantify the rate of thromboembolic events (TE) in pregnant women with SARS-CoV-2 infection and to characterize risk factors to provide a basis for individualized recommendation on prophylactic measures.

**Methods:**

CRONOS is a multicenter, prospective observational study conducted in Germany and Austria during the COVID-19 pandemic. Pregnant women with confirmed SARS-CoV-2 infection were enrolled. Data on demographics, medical history, COVID-19-related aspects, and pregnancy and birth outcomes were collected. TE was particularly queried and used as the primary outcome. A combination of “TE,” “maternal or fetal death,” or “severe postpartum hemorrhage” was defined as a secondary endpoint. Risk analyses were performed using univariate and multivariable logistic regression models.

**Results:**

Data from 8033 pregnant patients showed 40 TEs (0.5% incidence). TE rates were 10% in ICU patients, 0.2–0.4% in those with moderate-to-mild COVID-19, and < 0.1% in asymptomatic women. Pulmonary embolism occurred in 21 cases, deep vein thrombosis in 12, and 7 had atypical or arterial TE. Risk factors included advanced gestational age, COVID-19 symptoms, hospitalization or ICU admission, premature birth, cesarean section, delivery within 4 weeks of infection, higher weight gain, anemia, and chronic inflammatory bowel disease. COVID-19 vaccination reduced risk. The logistic risk model yielded an AUC of 0.87 (95% CI 0.81–0.94).

**Conclusion:**

The TE rate in pregnant women is largely determined by the severity of the disease. In asymptomatic or mild cases, other factors outweigh TE risk, while severe COVID-19 requiring ICU admission poses a high TE risk despite prophylaxis.

## What does this study add to the clinical work

**Table:** 

This study highlights that the risk of thromboembolic events (TE) in pregnant women with SARS-CoV-2 infecton is strongly linked to disease severity, with ICU admission posing the highest risk despite prophylaxis. It underscores the need for individualized risk stratification in clinical practice, considering factors like vaccination status, gestational age, and pre-existing conditions to guide anticoagulation therapy.	

## Introduction

The coronavirus disease COVID-19, which is caused by the “severe acute respiratory syndrome coronavirus 2” (SARS-CoV-2), primarily affects the respiratory system, leading to acute respiratory distress syndrome but is also considered a multi-organ disease. In this context, thromboembolic events (TE) have been considered a challenging problem [1, 2], particularly in pregnancy and puerperium [3, 4]. Pregnancy per se fulfills the criteria of Virchow’s triad, which is characterized by physiological hypercoagulability due to increased production of coagulation factors and a decrease in fibrinolytic activity, venous stasis and birth trauma. The absolute incidence of TE in pregnant women is estimated at 0.1% [5], and the daily risk is 5–10 times higher than in non-pregnant women [6, 7].

Systemic infections were considered additional transient risk factors for TE [8]. Particularly, patients with severe COVID-19 are at risk for thromboembolic complications through at least two different mechanisms: immunothrombogenic processes and severe illness with the need of hospitalization and treatment [9]. Immunothrombosis is a complex reciprocal process linking inflammation and coagulation [10, 11]. The main components of immunothrombosis are endothelial activation/dysfunction as a direct endothelial response to the virus, inflammation with activation of monocytes/macrophages, a massive increase in the production of proinflammatory cytokines (“cytokine storm”), activation of the coagulation cascade and finally hypoxia, which subsequently lead to micro- and macrovascular thrombosis [12–14]. Hospitalization-associated bedrest/immobilization additionally contributes to thromboembolic risk. In pregnancy, the risk of hospitalization and ICU admission for COVID-19 increases with ongoing gestational age [8, 9, 12–15].

As there was hardly any specific data available at the beginning of the pandemic, the “COVID-19-Related Obstetric and Neonatal Outcome Study” (CRONOS), a nationwide, multicenter, prospective observational study involving more than 8000 women with SARS-CoV-2 infection during pregnancy from 130 hospitals, aimed to collect data on maternal and perinatal morbidity and mortality [15, 16]. TE—among others—was considered an important research question not only for a better understanding of the disease, but also for evidence-based practices to protect maternal health and mitigate the negative consequences of TE associated with COVID-19 and pregnancy in future. The aim of this project was to determine the rate of TE in women affected by SARS-CoV-2 infection during their pregnancy and to correlate these data with disease severity, virus variants, immunization status and other risk factors.

## Materials and methods

CRONOS is a multicentric prospective observational study established by the German Society of Perinatal Medicine in April 2020 with the aim of offering a timely and fact-based extension of the counseling of pregnant women. Women with confirmed SARS-CoV-2 infection at any time during pregnancy who were treated in one of the participating maternity clinics regardless of the indication were included. Ethics approval was obtained (University Hospital Schleswig–Holstein, Kiel, AZ: D 451/20) and supplemented by votes from the local ethics committees. Information on CRONOS is published on the website www.dgpm-online.org and in the German Register of Clinical Studies (DRKS00021208). Methods and parts of the study results have been published [15–20].

### Data management

For collecting data, a reporting form was developed using the cloud-based electronic data capture platform (castoredc.com, Amsterdam, Netherlands). According to the study protocol, all women were prospectively enrolled in the study at first presentation in the maternity hospitals. Informed consent was obtained. Information on demographic characteristics, comorbidities, previous and current pregnancy characteristics, COVID-19-associated symptoms and treatments, pregnancy and birth specific events, and neonatal outcomes were entered. Specifically, the case report form asked for TE in the context of COVID-19. If indicated, the CRONOS study center sent a detailed questionnaire to the reporting hospital for further information. In addition to women with confirmed TE, women who were specifically treated for suspected TE were also counted.

### Study cohort

**Fig. 1:**
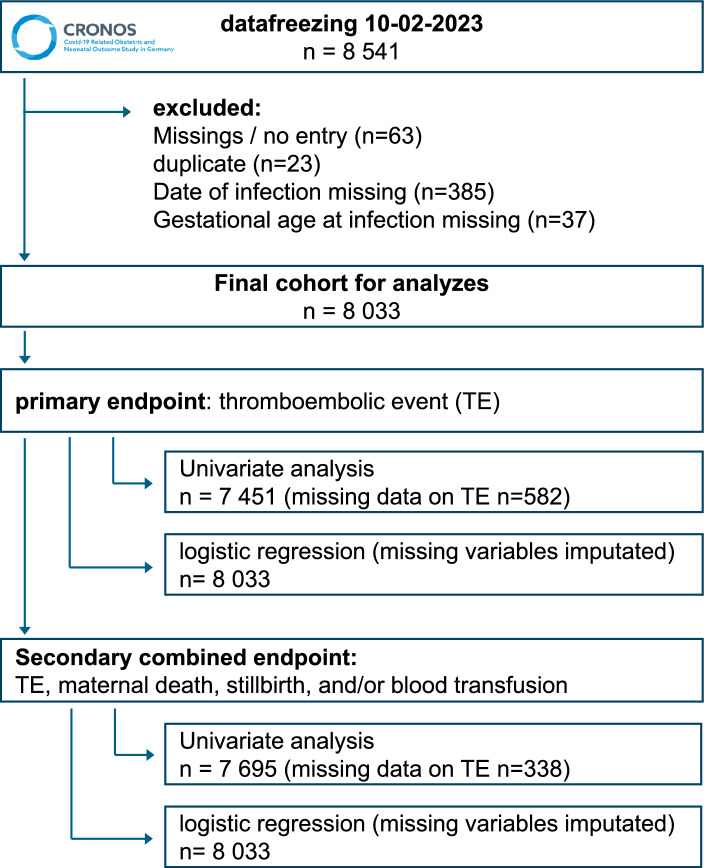
Flowchart of data cleaning and analysis steps. The initial dataset included 8541 cases, with exclusions due to missing entries, duplicate cases, and missing infection or gestational age data, resulting in a final cohort of 8033 cases. The figure illustrates the process for analyzing the primary endpoint (TE incidence) and the secondary combined endpoint (TE, maternal death, stillbirth, and/or blood transfusion)

At the time point of data extraction, obstetricians and neonatologists from 130 maternity hospitals had actively provided data to CRONOS. 8541 extracted cases from April 3, 2020, to December 31, 2022, underwent review and plausibility check (Fig. 1).

### Definition of primary and secondary end points

The primary outcome was defined as any venous or arterial TE including microthromboembolic events and disseminated intravascular coagulopathy (DIC) following SARS-CoV-2 infection in pregnancy. A combination of adverse events (CAE) that may be associated with the coagulation system were chosen as secondary endpoint. These were fulfilled if one of the following events were indicated: TE, blood transfusion for peripartum hemorrhage (PPH), maternal death or fetal death in the context of COVID-19 or timely related (within 4 weeks) to SARS-CoV-2 infection.

### Statistical analysis

Categorical variables were summarized as absolute (*n*) and relative frequencies (%). Continuous data were shown as mean (M) and standard deviation (SD). Univariate analyses were performed with Mann–Whitney *U* tests and Chi-square resp. Fisher’s exact test were first employed to evaluate bivariate risk factors for primary and secondary endpoints. Fisher’s exact test is used as an alternative to the Chi-square test when at least 20% of the cells have an expected frequency of less than 5. Odds ratio (OR) and corresponding 95% confidence interval (CI) were shown. Subgroup analyses and adjustment for anticoagulant medication was performed.

Significant covariates in bivariate testing were included in a backward stepwise logistic regression model to identify independent risk factors for thrombosis and the combined endpoint incorporating thromboembolic event, maternal death, stillbirth at more than 22 + 0 weeks or blood transfusion for PPH timely related to SARS-CoV-2 infection. Receiver operating characteristic (ROC) analyses were performed to receive the area under the curve (AUC) incl. 95% CI as an overall measure of model fit. In addition, Nagelkerke’s R2 was used as a measure of model fit. Multiple logistic regression was applied to the original dataset as well as on the imputed dataset. Within regression modeling incorporating predictor variables, only complete cases can be used. To analyze the impact of reducing the sample size to complete cases, sensitivity analyses using imputation strategies were applied. Therefore, missing values were imputed using mean/median imputation for continuous variables. For categorical variables, either a separate category was defined, or missing value category was summarized with a pre-defined variable value following worst-case strategy. *p* < 0.05 was considered statistically significant. All analyses were conducted using R with R studio (version 2022.02.0).

## Results

### Cohort description

Patients’ characteristics of the full cohort with 8033 pregnant patients are presented in Table 1; 223 women (2.8%) suffered from severe COVID-19 requiring ICU admission. Eleven women died (0.1%). In total, 40 TEs (incidence 0.5%) were reported.

**Table 1:** Patients’ characteristics of the full cohort

	Cohort					Missing		
	*n*	(%)	[95%CI]		*n*	(%)		[95%CI]
Number	8033							
Baseline characteristics								
Maternal age > 35 years at booking	1683	(21.0)	[20.1; 21.9]		37	(0.5)		[0.3; 0.6]
Primiparity	3102	(38.9)	[37.6; 39.7]		61	(0.8)		[0.6; 0.1]
Multiparity > 2	723	(9.0)	[8.4; 9.6]		61	(0.8)		[0.6; 0.1]
Multiple pregnancy	239	(3.0)	[2.6; 3.4]		76	(0.9)		[0.8; 1.2]
Arteficial reproductive therapy (ART)	292	(3.6)	[3.2; 4.1]		959	(11.9)		[11; 13]
Maternal BMI (kg/m^2^) ≥ 30 at booking	1408	(17.5)	[16.7; 18.4]		694	(9.6)	[8.1; 9.3]	
Weight gain per week > 0.5 kg (from booking to week of infection)	857	(10.7)	[10.0; 11.4]		2381	(29.6)	[29; 31]	
Ethnicity (in % North European)	4152	(51.7)	[50.6; 52.8]		1266	(15.8)	[15.0; 17]	
Limited communication	938	(11.7)	[11.0; 12.4]		49	(0.6)	[0.5; 0.8]	
Smoking before pregnancy	691	(8.6)	[8.0; 9.2]		1257	(15.6)	[16; 17]	
Smoking during pregnancy	333	(4.1)	[3.7; 4.6]		240	(3.0)	[2.6; 3.4]	
Concomittant disease	2850	(35.5)	[34; 36.5]		149	(1.9)	[1.6; 2.2]	
Minor concomittant disease	1946	(24.2)	[23; 25.2]					
Concomittant disease with regular medical treatment	779	(9.7)	[9.1; 10.4]					
Concomittant disease with frequent visites to the doctor	125	(1.6)	[1.3; 1.9]					
Cardiovascular disease, e. g. high blood pressure	483	(6.0)	[5.5; 6.6]					
Anemia	255	(3.2)	[2.8; 3.6]					
Heriditary thrombophilia or previous thromboembolic event	207	(2.6)	[2.3; 2.9]					
Diabetes mellitus type 1 or type 2	82	(1.0)		[0.8; 1.3]				
Autoimmune disease like SLE or RA	80	(1.0)		[0.8; 1.2]				
Inflammatory bowel disease	49	(0.6)		[0.5; 0.8]				
Chronic kidney disease or proteinuria	33	(0.4)		[0.3; 0.6]				
SARS-CoV-2 infection related aspects								
Weeks of gestation at SARS-CoV-2 infection								
1st trimester SARS-CoV-2 infection	845	(10.5)	[9.9; 11.2]		0.0	(0.0)		[0.0; 0.0]
2nd trimester SARS-CoV-2 infection	2274	(28.3)	[27; 29.3]		0.0	(0.0)		[0.0; 0.0]
3rd trimester SARS-CoV-2 infection	4914	(61.2)	[60; 62.2]		0.0	(0.0)		[0.0; 0.0]
Virus variant of concern (VOC)								
Wild type	2416	(30.1)	[29.1; 31.1]		0.0	(0.0)		[0.0; 0.0]
Alpha	684	(8.5)		[7.9; 9.1]	0.0	(0.0)		[0.0; 0.0]
Delta	1543	(19.2)		[18.4;20.1]	0.0	(0.0)		[0.0; 0.0]
Omicron	3390	(42.2)		[41.1;43.3]	0.0	(0.0)		[0.0; 0.0]
Vaccination against COVID-19 before SARS-CoV-2 infection	2156	(26.8)	[26;27.8]		697	(8.7)		[8.1; 9.3]
Severity Score of SARS-CoV-2 infection								
Asymptomatic SARS-CoV-2 infection	1364	(17.0)		[16.2;17.8]	643	(8.0)		[7.4; 8.6]
Mild COVID-19	3305	(41.1)		[40.1;42.2]	643	(8.0)		[7.4; 8.6]
Moderate COVID-19 (fever or dyspnea)	2498	(31.1)		[30.1;32.1]	643	(8.0)		[7.4; 8.6]
Severe COVID-19 (requiering intensive care monitoring)	223	(2.8)		[2.4;3.2]	643	(8.0)		[7.4; 8.6]
Symptomatic disease (COVID-19)	6026	(75.0)	[74.1; 76.0]		643	(8.0)		[7.4; 8.6]
Fever	2007	(25.0)		[24.0; 25.9]				
Cough	3757	(46.8)		[45.7; 47.9]				
Dyspnea	1410	(17.6)		[16.7; 18.4]				
Fatigue	2934	(36.5)		[35.5; 37.6]				
Outpatient at SARS-CoV-2 infection	3763	(46.8)	[46; 47.9]		288	(3.6)		[3.2; 4.0]
Admission to hospital within 4 weeks after infection	3453	(43.0)	[42; 44.1]		286	(3.6)		[3.2; 4.0]
COVID-19 as a reason for admission to hospital	828	(10.3)	[9.7; 11.0]		264	(3.3)		[2.9; 3.7]
Maternal treatment or monitoring for COVID-19	839	(10.4)		[9.8; 11.1]	250	(3.1)		[2.8; 3.5]
Oxygen treatment	364	(4.5)		[4.1; 5.0]	265	(3.3)		[2.9; 3.7]
Intensive care monitoring	223	(2.8)		[2.4; 3.2]	267	(3.3)		[3.0; 3.7]
Invasive ventilation	107	(1.3)		[1.1; 1.6]	270	(3.4)		[3.0; 3.8]
Thromboembolic event in the context of COVID-19	40	(0.5)	[0.4; 0.7]		582	(7.2)		[6.7; 7.8]
Maternal death	11	(0.1)	[0.1; 0.2]		272	(3.4)		[3.0; 3.8]
Anticoagulation								
Anticoagulation treatment before SARS-CoV-2 infection	167	(2.1)	[1.8; 2.4]		18	(0.2)		[0.1; 0.4]
Initiation of anticoagulation treatment with SARS-CoV-2 infection	2303	(28.7)	[28; 29.7]		782	(9.7)		[9.1; 10]
Reason for anticuagulation treatment^a^								
Other than COVID-19	458	(20.3)	[18.7; 22.0]		63	(2.7)		[2.1; 3.5]
COVID-19, no other risks	1353	(60.1)	[58.0; 62.1]		63	(2.7)		[2.1; 3.5]
COVID-19 and other moderate risks	395	(17.5)	[16.0; 19.2]		63	(2.7)		[2.1; 3.5]
COVID-19 and high risk for thromboembolic events	47	(2.1)	[1.6; 2.8]		63	(2.7)		[2.1; 3.5]
Perinatal outcome	7298	(90.9)	[90; 91.5]		735	(9.1)		[8.5; 9.8]
Abortion (delivery < 22 weeks of gestation; infection < 22 weeks of gestation)	57	(3.0)	[2.3; 3.9]		270	(14.2)		[12.7; 15.8]
Delivery ≥ 22 weeks of gestation among all women	7241	(90.1)	[90; 90.8]		735	(9.1)		[8.5; 9.8]
Preterm birth (≥ 22 and < 37 weeks of gestation)	897	(12.3)	[11.6; 13.1		0	(0.0)		[0.0; 0.1]
Preterm birth (≥ 22 and < 37 weeks of gestation) within 4 weeks of infection	554	(7.6)	[7.0; 8.2]		0	(0.0)		[0.0; 0.1]
Delivery ≥ 22 weeks of gestation within 4 weeks of infection	3253	(44.9)	[43.7; 46.0]		0	(0.0)		[0.0; 0.1]
Cesarean section as the mode of delivery	2555	(34.8)	[33.7; 35.9]		40	(0.5)		[0.4; 0.7]
Cesarean section within 4 weeks of infection	1245	(17.0)	[16.1; 17.8]		40	(0.5)		[0.4; 0.7]
Peripartum hemorrhage with estimated blood loss ≥ 1500 mL	103	(1.3)	[1.1; 1.6]		338	(4.4)		[4.0; 4.9]
Blood transfusion for peripartum hemorrhage	31	(0.4)	[0.3;0.6]		339	(4.4)		[4.0; 4.9]
Stillbirth (≥ 22 weeks of gestation)	58	(0.8)	[0.6;1.0]		27	(0.4)		[0.3; 0.5]
Stillbirth (≥ 22 weeks of gestation) within 4 weeks of infection	47	(0.6)	[0.5;0.9]		27	(0.4)		[0.3; 0.5]

Presented are frequencies and percentages with 95% confidence intervals for each variable and for missing values

^a^as judged by the attending obstetrician / physician

In the first phases of the pandemic (wild-type virus variant; February 2020 to February 2021), the incidence for TE was 0.4%. This was followed by 0.6% with the Alpha virus variant (March to May 2021), and 1.2% with Delta virus (June 2021 to December 2021). The incidence subsequently fell to 0.3% in the phase with dominance of Omicron virus variant (January 2022 to May 2022). Overall, TE-incidence in non-vaccinated women was 0.68% versus 0.23% in vaccinated women (*p* = 0.0221).

About one in ten women with severe COVID-19 who had to be monitored and treated in the ICU developed a TE (10%; 22 of 223 women). Nine women with TE had moderate COVID-19 and eight women had mild COVID-19, corresponding to an incidence of 0.36% and 0.24%, respectively. Only one woman with asymptomatic infection suffered from TE (0.07% of all 1364 women with asymptomatic SARS-CoV-2 infection). The median interval between diagnosis of SARS-CoV-2 infection and the diagnosis of TE was 1 week (interquartile range (IQR) 0 to 2 weeks).

In more than half of the women (*n* = 22), diagnosis of TE was made postpartum and on median at day 7 (IQR 4 to 12 days). In the 15 women who were diagnosed with TE during pregnancy, the diagnosis was made on median in 30 weeks of gestation (WOG; IQR 24 to 36). In seven cases, exact information about the time of TE diagnosis was missing. Pulmonary embolism (PE) was diagnosed in 21 women (0.26%), lower extremity deep vein thrombosis (LE-DVT) was observed in 12 women (0.15%), and 7 women (0.09%) had either atypical venous TE or arterial TE. Three women had a combination of PE and/or DVT and/or atypical TE. In three women, information on the location of the TE was not provided. Twenty-four women developed TE despite receiving anticoagulation at the onset of SARS-CoV-2 infection. Five women died.

### Primary endpoint: TE

For univariate analyses, 7451 datasets were available with data on TE given (missing data *n* = 582). The Alpha and Delta virus variant were more likely to be associated with TE than the wild-type or Omicron variant (OR = 3.2, 95% CI 1.7–6.0). Likewise, symptomatic infection increased the risk when compared to asymptomatic infection (OR = 8.4, 95% CI 1.2–61.2), and this was highest in women with severe COVID-19 (Table 2). Fever, dyspnea, and cough, but not fatigue was associated with TE. The risk for TE increased with admission to hospital for COVID-19. The need of breathing support and ICU monitoring for COVID-19 further increased the risk and was highest for women who required invasive ventilation. Vaccination against COVID-19 reduced the risk for TE by 60% (Table 2). Giving birth, especially preterm and by cesarean section increased the risk for TE. Peripartum hemorrhage was not identified as a risk factor even if a blood transfusion was given. Among concomitant conditions, increased weight gain during pregnancy (> 0.5 kg/week), anemia, and chronic inflammatory bowel disease were identified as risk factors. Women having difficulties to communicate with their health care providers due to foreign language were on higher risk (Table 2).

**Table 2:** Univariate analysis for the primary endpoint “thromboembolism” (TE) for each variable is displayed with frequencies for women suffering (TE) and not suffering (No TE) from TE

		No TE (*n*)	TE (*n*)	Chi-square	df	*p*-value	OR	95%CI
COVID-19 related factors								
WOG at SARS-CoV-2 infection	0–20	1611	4	8.67	2	**0.013**		
	21–36	3835	30				3.2	1.11–8.96
	≥ 37	1965	6				1.2	0.35–4.37
SARS-CoV-2 variant (VoC)	wild-type	2175	9	18.45	3	** < 0.001**		
	Alpha	627	4				1.5	0.47–5.02
	Delta	1419	18				3.1	1.37–6.84
	Omicron	3190	9				0.7	0.27–1.72
Vaccination against COVID-19 (any vaccine dose)	No	4755	32	4.83	1	**0.028**		
	Yes	2049	5				0.4	0.14–0.93
Severity of COVID-19	Asymptomatic	1210	1	377.13	3	** < 0.001**		
	Mild COVID-19	3097	8				3.1	0.39–25.02
	Moderate COVID-19	2337	9				4.7	0.59–36.82
	Severe COVID-19	183	22				145.5	19.49–1085.69
Fever	No	4170	11	23.20	1	** < 0.001**		
	Yes	1856	24				4.9	2.40–10.03
Cough	No	2719	7	8.59	1	**0.003**		
	Yes	3497	29				3.2	1.41–7.36
Dyspnea	No	5217	13	46.42	1	** < 0.001**		
	Yes	1292	24				7.5	3.79–14.68
Fatigue	No	3190	11	2.89	1	0.089		
	Yes	2750	18				1.9	0.90–4.03
Outpatient treatment at the time of infection	No	3690	27	5.97	1	**0.015**		
	Yes	3518	11				0.4	0.21–0.86
Admission to hospital within 4 weeks after infection	No	4180	8	21.67	1	** < 0.001**		
	Yes	3092	31				5.2	2.40–11.41
Admission to hospital because of COVID-19	No	6551	16	101.50	1	** < 0.001**		
	Yes	725	23				13.0	6.83–24.70
Any hospital treatment for COVID-19	No	6630	13	141.14	1	** < 0.001**		
	Yes	742	27				18.6	9.53–36.12
Initiation of oxygen treatment for COVID-19	No	7058	18	241.34	1	** < 0.001**		
	Yes	308	22				28.0	14.87–52.76
Admission to ICU for COVID-19	No	7184	18	407.73	1	** < 0.001**		
	Yes	183	22				48.0	25.30–90.99
Invasive ventilation for COVID-19	No	7286	22	593.82	1	** < 0.001**		
	Yes	79	18				75.5	38.95–146.17
Obstetric events timely related to COVID-19 (within 4 weeks after SARS-CoV-2 infection)								
Birth	No	3988	10	16.64	1	** < 0.001**		
	Yes	3004	30				4.0	1.94–8.16
Preterm birth	No	6111	17	71.57	1	** < 0.001**		
	Yes	881	23				9.4	4.99–17.64
Stillbirth ≥ 22 + 0 weeks of gestation	No	6926	39	2.44	1	0.118		
	Yes	41	1				4.3	0.58–32.28
Cesarean section (CS)	No	5842	19	38.28	1	** < 0.001**		
	Yes	1128	21				5.7	3.07–10.68
Peripartum hemorrhage	No	6679	40	0.58	1	0.448		
	Yes	96	0				na	
Blood transfusion because of peripartum hemorrhage	No	6745	40	0.17	1	0.678		
	Yes	29	0				na	
Concomitant factors not related to COVID-19								
Higher maternal age (> 35 years)	No	5813	33	0.35	1	0.556		
	Yes	1575	7				0.8	0.35–1.77
Communication without any language barriers	No	938	9	3.99	1	**0.046**		
	Yes	6384	29				0.5	0.22–1.00
Multiparity (> 2 births prior to the current pregnancy)	No	6717	33	3.50	1	0.061		
	Yes	664	7				2.1	0.95–4.87
Medically assisted reproduction (MAR)	No	6408	32	0.11	1	0.743		
	Yes	279	1				0.7	0.10–5.27
Multiple pregnancy	No	7148	37	2.83	1	0.092		
	Yes	218	3				2.7	0.81–8.69
Maternal obesity at booking (BMI ≥ 30 kg/m^2^)	No	5550	26	0.08	1	0.771		
	Yes	1320	7				1.1	0.49–2.61
Maternal average weight gain > 0.5 kg per week of gestation	No	4470	14	5.18	1	**0.023**		
	Yes	811	7				2.8	1.11–6.85
Former smoker (smoking before pregnancy)	No	5683	30	1.65	1	0.199		
	Yes	647	1				0.3	0.04–2.15
Current smoker (during pregnancy)	No	6959	35	1.55	1	0.213		
	Yes	308	0				na	
Concomitant maternal disease (severity)	No	4672	23	1.58	3	0.664		
	Mild	1825	9				1.0	0.46–2.17
	Moderate	729–	6				1.7	0.68–4.12
	Severe	116	1				1.8	0.23–13.08
Heart disease or hypertension preeclampsia / HELLP	No	6880	37	0.09	1	0.763		
	Yes	463	2				0.8	0.19–3.34
Diabetes mellitus (preexistent)	No	7271	39	0.39	1	0.531		
	Yes	73	0				na	
Anemia (Hb < 10.5 g/dL or as indicated by health care provider)	No	7090	33	11.56	1	** < 0.001**		
	Yes	239	5				4.5	1.74–11.61
Chronic inflammatory bowel disease	No	7247	37	12.07	1	** < 0.001**		
	Yes	46	2				8.5	1.99–36.38
Chronic kidney disease	No	7309	39	0.18	1	0.675		
	Yes	33	0				na	
Autoimmunic disease (SLE, APL, RA)	No	7266	38	0.88	1	0.349		
	Yes	76	1				2.5	0.34–18.56
Former thrombotic disease or known thrombophilia	No	7147	37	0.90	1	0.343		
	Yes	196	2				2.0	0.47–8.24
Former thrombotic disease or known thrombophilia (low-risk / high-risk)	No	7166	37	1.80	3	0.615		
	Low risk	59	1				3.3	0.44–24.32
	High risk	51	0				na	
Anticoagulation at time of infection								
Initiation of anticoagulation treatment before or with SARS-CoV-2 infection	No	4724	14	17.56	1	** < 0.001**		
	Yes	2278	25				3.7	1.92–7.14
Reason for anticoagulation treatment as indicated by the attending obstetrician / physician	Other indication than COVID-19	439	5	59.37	3	** < 0.001**		
	COVID-19 only	1265	11				0.8	0.26–2.21
	COVID-19 and moderate risks	378	3				0.7	0.17–2.94
	COVID-19 and severe risks	39	6				13.5	3.94–46.27

Odds ratios are presented with 95% confidence intervals (CI)

*na* not applicable due to zero frequencies

Significant effects are highlighted with *p*-values in bold

WoG Weeks of gestation; ICU Intensive care unit; SLE Systemic luous erythematodes; APL Antiphospholipid syndrome; RA Rheumatic arthritis

The identified risk factors were included in a logistic regression model. A higher average weight gain per week of pregnancy until SARS-CoV-2 infection, premature birth within 4 weeks of SARS-CoV-2 infection and severe COVID-19, i.e., admission to intensive care and invasive ventilation, independently increased the risk of TE. Moreover, chronic inflammatory bowel disease revealed an independent risk factor. A longer time period between SARS-CoV-2 infection and first contact with the hospital was associated with a reduced risk (Table 3).

**Table 3:** Multivariate logistic regression model for the primary endpoint thromboembolic events (TE)

		Reg. coeff. B	SE	Wald	df	*p*-value	OR	95%CI
Severity of COVID-19	Asymptomatic			16.1	3	0.00		
	Mild COVID-19	1.3	1.1	1.5	1	0.22	3.8	[0.5–30.6]
	Moderate COVID-19	1.9	1.1	3.0	1	0.08	6.4	[0.8–51.0]
	Severe COVID-19	3.6	1.1	10.1	1	0.00	36.3	[4.0–332.5]
Invasive ventilation for COVID-19		1.4	0.6	5.6	1	0.02	4.0	[1.3–12.8]
Maternal average weight gain per week of gestation until SARS-CoV-2 infection	kg/week	0.4	0.2	3.9	1	0.05	1.4	[1.0–2.0]
Chronic inflammatory bowel disease		2.6	0.9	8.5	1	0.00	13.9	[2.4–81.5]
Time to first contact with the clinic after infection	Weeks	-0.1	0.0	3.9	1	0.05	0.9	[0.8–1.0]
Preterm birth within 4 weeks after SARS-CoV-2 infection(1)		1.0	0.4	6.1	1	0.01	2.7	[1.2–6.1]
Constant		-7.4	1.0	53.1	1	0.00	0.0	

			SE			Asympt. sign	AUC	95%CI
ROC analysis			0.034			0	0.872	[0.806–0.939]

A backward stepwise logistic regression model was used to identify independent risk factors. Variables of the final model are shown with regression coefficients. Receiver operating characteristic (ROC) analyses were performed to receive the area under the curve (AUC) incl. 95% confidence intervals (CI)

### Secondary endpoint: CAE

For analyses of CAE, 7695 datasets were available with 131 (1.7%) women presenting with at least one of the defined events (missing data *n* = 338). Risk factors associated with CAE were like those of the primary endpoint (Table 4). Identified risk factors were included in a logistic regression model. The virus variant (Alpha/Delta), severity of COVID-19, invasive ventilation, birth within 4 weeks after SARS-CoV-2 infection, especially when preterm, and performed by cesarean section, but also chronic inflammatory bowel disease, and initiation of anticoagulation treatment were independently associated with CAE (Table 5).

**Table 4:** Univariate analysis for the secondary endpoint, the combined endpoint incorporating thromboembolic event, maternal death, stillbirth at more than 22+0 weeks or blood transfusion for PPH timely related to SARS-CoV-2 infection (CAE)

		No CAE (*n*)	CAE (*n*)	Chi-square	df	*p*-value	OR	95%CI
COVID-19 related factors								
WOG at SARS-CoV-2 infection	0–20	1634	16	14.28	2	** < 0.001**		
	21–36	3907	88				2.30	1.35–3.93
	≥ 37	2154	27				1.28	0.69–2.38
SARS-CoV-2 variant (VoC)	wild-type	2337	33	29.00	3	** < 0.001**		
	Alpha	645	20				2.20	1.25–3.85
	Delta	1451	43				2.10	1.33–3.32
	Omicron	3262	35				0.76	0.47–1.23
Vaccination against COVID-19 (any vaccine dose)	No	4954	103	11.33	1	** < 0.001**		
	Yes	2080	19				0.44	0.27–0.72
Severity of COVID-19	Asymptomatic	1345	19	307.93	3	** < 0.001**		
	Mild COVID-19	3171	31				0.69	0.39–1.23
	Moderate COVID-19	2380	36				1.07	0.61–1.87
	Severe COVID-19	185	37				14.16	7.97–25.14
Fever	No	4368	56	21.37	1	** < 0.001**		
	Yes	1892	57				2.35	1.62–3.41
Cough	No	2874	44	1.41	1	0.235		
	Yes	3579	69				1.26	0.86–1.84
Dyspnea	No	5421	75	22.09	1	** < 0.001**		
	Yes	1323	44				2.40	1.65–3.50
Fatigue	No	3366	54	0.11	1	0.740		
	Yes	2800	48				1.07	0.72–1.58
Outpatient treatment at the time of infection	No	3855	94	22.83	1	** < 0.001**		
	Yes	3606	35				0.40	0.27–0.59
Admission to hospital within 4 weeks after infection	No	4216	26	67.01	1	** < 0.001**		
	Yes	3313	104				5.09	3.30–7.84
Admission to hospital because of COVID-19	No	6771	90	58.05	1	** < 0.001**		
	Yes	763	40				3.94	2.70–5.77
Any hospital treatment for COVID-19	No	6858	86	75.00	1	** < 0.001**		
	Yes	782	44				4.49	3.10–6.50
Initiation of oxygen treatment for COVID-19	No	7311	92	180.83	1	** < 0.001**		
	Yes	322	38				9.38	6.32–13.91
Admission to ICU for COVID-19	No	7449	93	312.01	1	** < 0.001**		
	Yes	185	37				16.02	10.65–24.09
Invasive ventilation for COVID-19	No	7555	100	457.88	1	** < 0.001**		
	Yes	77	30				29.44	18.48–46.89
Obstetric events timely related to COVID-19 (within 4 weeks after SARS-CoV-2 infection)								
Birth	No	3977	21	80.87	1	** < 0.001**		
	Yes	3190	110				6.53	4.09–10.44
Preterm birth	No	6296	48	296.85	1	** < 0.001**		
	Yes	871	83				12.50	8.70–17.96
Stillbirth ≥ 22 + 0 weeks of gestation	No	7140	84	2578.35	1	** < 0.001**		
	Yes	0	47				na	
Cesarean section (CS)	No	5942	85	28.55	1	** < 0.001**		
	Yes	1200	45				2.62	1.82–3.78
Peripartum hemorrhage	No	6764	97	490.83	1	** < 0.001**		
	Yes	71	32				31.43	19.78–49.93
Concomittant factors not related to COVID-19								
Higher maternal age (> 35 years)	No	6045	104	0.02	1	0.880		
	Yes	1622	27				0.97	0.63–1.48
Communication without any language barriers	No	1004	30	11.03	1	** < 0.001**		
	Yes	6593	99				0.50	0.33–0.76
Multiparity (> 2 births prior to the current pregnancy)	No	6954	114	2.33	1	0.127		
	Yes	696	17				1.49	0.89–2.49
Medically assisted reproduction (MAR)	No	6506	111	0.14	1	0.713		
	Yes	283	4				0.83	0.30–2.26
Multiple pregnancy	No	7417	123	4.71	1	**0.030**		
	Yes	220	8				2.19	1.06–4.54
Maternal obesity at booking (BMI ≥ 30 kg/m^2^)	No	5704	90	1.07	1	**0.300**		
	Yes	1361	27				1.26	0.81–1.94
Maternal average weight gain > 0.5 kg per week of gestation	No	4624	76	0.03	1	0.864		
	Yes	833	13				0.95	0.52–1.72
Former smoker (smoking before pregnancy)	No	5874	100	1.51	1	0.220		
	Yes	663	7				0.62	0.29–1.34
Current smoker (during pregnancy)	No	7181	118	0.01	1	0.913		
	Yes	320	5				0.95	0.39–2.34
Concomitant maternal disease (severity)	No	4836	85	5.25	3	0.154		
	Mild	1884	23				0.69	0.44–1.10
	Moderate	742	18				1.38	0.83–2.31
	Severe	122	3				1.40	0.44–4.49
Heart disease or hypertension preeclampsia / HELLP	No	7118	115	4.78	1	**0.029**		
	Yes	467	14				1.86	1.06–3.26
Diabetes mellitus (preexistent)	No	7510	125	5.45	1	**0.020**		
	Yes	76	4				3.16	1.14–8.78
Anemia (Hb < 10.5 g/dl or as indicated by health care provider)	no	7324	122	0.80	1	0.370		
	Yes	247	6				1.46	0.64–3.34
Chronic inflammatory bowel disease	No	7486	126	5.87	1	**0.015**		
	Yes	46	3				3.87	1.19–12.62
Chronic kidney disease	No	7551	129	0.56	1	0.453		
	Yes	33	0				na	
Autoimmunic disease (SLE, APL RA)	No	7506	127	0.34	1	0.562		
	Yes	78	2				1.52	0.37–6.24
Former thrombotic disease or known thrombophilia	No	7383	126	0.06	1	0.813		
	Yes	202	3				0.87	0.27–2.76
Former thrombotic disease or known thrombophilia (low-risk / high-risk)	No	7401	127	0.91	3	0.823		
	Low risk	61	1				0.96	0.13–6.95
	High risk	53	0					
Anticoagulation at time of infection								
Initiation of anticoagulation treatment before SARS-CoV-2 infection	No	7516	128	0.21	1	0.646		
	Yes	163	2				0.72	0.18–2.94
Initiation of anticoagulation treatment with SARS-CoV-2 infection	No	4866	66	16.16	1	** < 0.001**		
	Yes	2217	61				2.03	1.43–2.88
Reason for anticuagulation treatment as indicated by the attending obstetrician / physician	other indication than COVID-19	446	12	79.68	3	** < 0.001**		
	COVID-19 only	1309	25				0.71	0.35–1.42
	COVID-19 and moderate risks	376	13				1.29	0.58–2.85
	COVID-19 and severe risks	36	11				11.36	4.68–27.54
Initiation of anticoagulation treatment before or with SARS-CoV-2 infection	No	4773	65	15.24	1	** < 0.001**		
	Yes	2333	63				1.98	1.40–2.81

For each variable frequencies for women suffering (CAE) and not suffering (No CAE) from CAE are displayed. Odds ratios are presented with 95% confidence intervals (CI)

Significant effects are highlighted with *p*-values in bold

WoG Weeks of gestation; ICU Intensive care unit; SLE Systemic luous erythematodes; APL Antiphospholipid syndrome; RA Rheumatic arthritis

**Table 5:** Multivariate logistic regression model for the secondary endpoint, the combined endpoint incorporating thromboembolic event, maternal death, stillbirth at more than 22+0 weeks or blood transfusion for PPH timely related to SARS-CoV-2 infection (CAE)

		Reg. coeff. B	SE	Wald	df	*p*-value	OR	95%CI
Virus variant (alpha/delta vs. wild-type/omicron)		0.5	0.2	6.6	1	0.010	1.7	1.1-2.4
Severity of COVID-19	Asymptomatic			12.6	3	0.005		
	Mild COVID-19	0.2	0.3	0.6	1	0.432	1.3	[0.7–2.2]
	Moderate COVID-19	0.7	0.3	4.9	1	0.028	1.9	[1.1–3.5]
	Severe COVID-19	1.6	0.5	9.8	1	0.002	4.9	[1.8–13.3]
Invasive ventilation for COVID-19		1.0	0.5	4.5	1	0.035	2.7	[1.1–7.0]
Initiation of anticoagulation treatment before or with SARS-CoV-2 infection		1.3	0.6	4.5	1	0.034	3.6	[1.1–12.0]
Chronic inflammatory bowel disease		2.0	0.7	8.1	1	0.004	7.2	[1.8–28.4]
Cesarean section within 4 weeks after infection		–1.2	0.3	19.4	1	< 0,001	0.3	[0.2–0.5]
Preterm birth within 4 weeks after infection		2.1	0.2	103.8	1	< 0,001	8.6	[5.7–12.9]
Constant		–7.7	0.7	128.6	1	< 0,001	0.0	

			SE			Asympt. sign	AUC	95%CI
ROC analysis			0.0172			0	0.868	[0.835–0.902]

A backward stepwise logistic regression model was used to identify independent risk factors. Variables of the final model are shown with regression coefficients. Receiver operating characteristic (ROC) analyses were performed to receive the area under the curve (AUC) incl. 95% confidence intervals (CI)

## Discussion

COVID-19 possesses a risk for thromboembolic complications through immunothrombogenic processes, inflammation and acute illness with the need of hospitalization and treatment [9–15]. It was assumed that this risk further increases in women with SARS-CoV-2 infection during their pregnancy. However, data for counseling pregnant women or for justifying anticoagulative measures and strategies on an individual basis were limited. Therefore, low molecular weight heparin has been increasingly prescribed universally as the pandemic continues [20]. The present study aimed at identifying risk factors and calculated the overall risk for TE to allow an individualized shared decision making. The incidence of TE ranged from 0.3% in the Omicron variant dominant phase to 1.2% in the Delta variant dominant phase. Disease severity was the main influencing factor, increasing the rate to 1 in 10 women admitted to the ICU due to COVID-19 compared to about 1 in 1000 in asymptomatic women. On top of this, invasive ventilation for COVID-19 was an independent risk factor. In addition to COVID-19-specific factors, increased average maternal weight gain, premature birth and chronic inflammatory bowel disease led to an increased risk. Neither diabetes, obesity or hypertension nor multiple pregnancies or assisted reproduction to achieve pregnancy, which usually pose a risk for TE [8, 21–25], were identified as risk factors. Vaccination reduced the risk by minimizing the severity of the disease. Administration of anticoagulation showed a paradoxical association with TE in the available data. This is best explained by the fact that the women at highest risk of TE, i.e., those with intensive monitoring, invasive ventilation and ECMO therapy, received anticoagulation but still developed TE, in line with reports of others [26–28].

In general, the incidence of venous TE in pregnant women is reported to be 0.1% [5]. More recent data on the incidence of thrombosis in pregnant women with COVID-19 from reviews and meta-analyses were based on data mostly from case series or smaller cohort studies and were reported at 0.27 to 0.48% [29–32]. Bruno et al. retrospectively analyzed insurance data from 2016 to 2020 with more than 800,000 deliveries, including 2432 with SARS-CoV-2 infection during pregnancy or up to 6 weeks postpartum. In their cohort, they found an incidence for TE of 1% in women with compared to 0.5% in those without SARS-CoV-2 infection [33]. Similarly, Ko et al. calculated the incidence of TE using a hospital-based database in the U.S. They considered data from March to September 2020 with approximately 500,000 women, 6550 had SARS-CoV-2 infection during delivery hospitalization. The incidence of TE was reported to be 0.3% in women with, and 0.1% in women without SARS-CoV-2 infection [34]. The overall low incidence in their study may be explained by the dominance of the wild-type virus variant at that time that less likely caused severe COVID-19 when compared to the Delta virus variant [35]. The different virus variants and the vaccination status have a significant impact on the severity of the disease with the Delta variant having the highest and the Omicron variants having the lowest severities [15, 35, 36]. In fact, the TE rate in the present study was rather related to the severity of the disease than the virus variant. Consistent with data presented here, Metz et al. found in a multicentric observational study with 1200 women with a positive SARS-CoV-2 test result and delivery between March 2020 and July 2020 a TE rate of 6% in severe/critically ill women compared to 0.2% in mild to moderate cases [37]. Of note, a disproportional prevalence of PE compared to DVT has been observed in general in COVID-19 patients [38] that may additionally determine disease progression. Data of Bruno et al. [33] and our own findings in pregnant women contribute, since as much as 50% of women with TE suffered from PE. Reasons for this may lie in the pathogenesis of focalized pulmonary immune thrombosis rather than thrombi embolized from the deep veins of the lower extremities [9].

The association of COVID-19 with TE in the general population has been reported soon after the COVID-19 outbreak and led to recommendations of anticoagulant prophylaxis [39]. Our study group recently reported that this recommendation was increasingly followed during the pandemic in Germany in women with peripartum SARS-CoV-2 infection [20]. In the present study, 29% received anticoagulant medication. In the context of severe COVID-19 recommendations are unambiguous. However, the question of when to prescribe prophylactic measures against TE in mild disease remains controversial. Most intervention studies in outpatients with mild COVID-19 have failed to demonstrate a benefit of low-dose prophylactic anticoagulation due to generally low event rates [40–43]. Unfortunately, pregnant women are often rather excluded from interventional trials because of safety concerns, yet on the same time are at higher risk than non-pregnant women of the same age for a variety of adverse events including severe COVID-19 and TE [44, 45]. Therefore, risk stratification based on individual risk factors for TE is recommended [46, 47]. The study results suggest that heparin should not be prescribed for low-risk patients with asymptomatic or mild SARS-CoV-2 infection but should be considered for women with severe COVID-19 or those with thrombophilia risk factors. Women with language barrier were more likely to suffer from TE. It remains speculative to assume why those were found disproportionally more often in the study group: a generally higher prevalence of infections [48], differing vaccine acceptance [49], or less frequent appointments to the physicians might be among those causes. This should sensitize healthcare providers to the need to target these women and provide them with the best possible information on health issues and merits further investigation.

Some limitations should be mentioned. Although 98% of all women in Germany visit a hospital at the latest at birth and the participating hospitals cover 30% of all births in Germany, a selection bias cannot be excluded. Therefore, the prevalence of TE in women with SARS-CoV-2 infection may be overestimated, as women with adverse events may be identified by the study sites more frequently. However, the registry allowed for inclusion of all women with SARS-CoV-2 infection noted in the pregnancy file. Regular testing for SARS-CoV-2 was mandatory during the study period in Germany at many occasions including visits to the doctor. Since pregnant women in Germany attend a doctor’s appointment at least once a month, they can be considered a highly observed population. Another limitation may be that, although the registry specifically asked about TEs related to COVID-19, some TEs may have been missed, particularly in the postpartum period, as only about one-third of the women were followed up for at least 4 weeks postpartum. However, for most women (> 70%), data from medical records were available for at least up to 4 weeks after SARS-CoV-2 infection and thus for the period considered as acute COVID-19 infection [50]. One strength of the study is the prospective nature of the registry and the quality of the data entry, which was ensured by weekly plausibility analyses and, in the case of data with a signal effect including TE, by additional queries to the reporting clinics [15]. Moreover, in this study, different virus variants and vaccination status were considered, while studies of others about TE in pregnancy in the context of COVID-19 mostly are limited to data of the wild-type pandemic phase in 2020 [31, 33, 34, 37].

In conclusion, pregnant women with COVID-19 have an increased risk of thromboembolic disease if they have severe symptoms. Additional risk factors contribute to the need for appropriate risk stratification using standardized risk assessment tools to be introduced into clinical practice. Good communication and collaboration between primary and secondary care, including midwifery staff in the community, is crucial especially for women with language barriers who are much more affected.

## Data Availability

No datasets were generated or analysed during the current study.
